# Visibility Evaluation of Fundic Gland Polyp Associated With Proton Pump Inhibitor in Texture and Color Enhancement Imaging

**DOI:** 10.1002/deo2.70147

**Published:** 2025-05-22

**Authors:** Ryota Uchida, Hiroya Ueyama, Tsutomu Takeda, Shunsuke Nakamura, Yasuko Uemura, Tomoyo Iwano, Momoko Yamamoto, Hisanori Utsunomiya, Daiki Abe, Shotaro Oki, Nobuyuki Suzuki, Atsushi Ikeda, Yoichi Akazawa, Kumiko Ueda, Mariko Hojo, Shuko Nojiri, Takashi Yao, Akihito Nagahara

**Affiliations:** ^1^ Department of Gastroenterology Juntendo University School of Medicine Tokyo Japan; ^2^ Medical Technology Innovation Center Juntendo University School of Medicine Tokyo Japan; ^3^ Department of Human Pathology Juntendo University Graduate School of Medicine Tokyo Japan; ^4^ Department of Pathophysiological Research and Therapeutics for Gastrointestinal Disease Juntendo University School of Medicine Tokyo Japan

**Keywords:** fundic gland polyp, fundic gland polyp associated with proton pump inhibitor, gray color sign, proton pump inhibitor, texture and color enhancement imaging

## Abstract

**Objectives:**

A ‘gray color sign’ (GCS) is a new endoscopic feature of fundic gland polyp associated with proton pump inhibitor (PPI‐FGP). Here, we compare the ability of texture and color enhancement imaging (TXI) to white light imaging (WLI) with regard to the detection of GCS.

**Methods:**

In this prospective study, 19 consecutive patients with PPI‐FGP were enrolled at our hospital from April 2021 to October 2022. Endoscopic images of PPI‐FGP using WLI, TXI mode1 (TXI‐1), TXI mode2 (TXI‐2), and narrow‐band imaging (NBI) were collected and compared by 10 endoscopists. Visibility of GCS by each mode (Image enhancement endoscopy) was scored as follows: 5, improved; 4, somewhat improved; 3, equivalent; 2, somewhat decreased; and 1, decreased. The inter‐rater reliability (intra‐class correlation coefficient, ICC) was also evaluated. The images were objectively evaluated based on L* a* b* color values and the color difference (ΔE*) in the CIE LAB color space system.

**Results:**

Improved visibility of GCS compared with WLI was achieved for: TXI‐1: 82.6%, TXI‐2: 86.9%, and NBI: 0% for all endoscopists. Total visibility scores were: TXI‐1, 44.9; TXI‐2, 42.9; NBI, 17.4 for all endoscopists. Visibility scores were significantly higher using TXI‐1 and TXI‐2 compared with NBI (*p* < 0.01). The inter‐rater reliability for TXI‐1 and TXI‐2 was “excellent” for all endoscopists. The use of ΔE* revealed statistically significant differences between WLI and TXI‐1 (*p* < 0.01).

**Conclusions:**

TXI is an improvement over WLI for the visualization of GCS, and can be used by both trainee and expert endoscopists with equal efficiency and accuracy.

## 1 Introduction

Proton pump inhibitors (PPIs) and potassium‐competitive acid blockers (PCABs) are used worldwide to treat acid‐related illnesses such as gastroesophageal reflux disease and peptic ulcers; they are also used to prevent accidental antithrombotic drug injury [1]. However, long‐term administration of PPIs and PCABs can trigger the development of new fundic gland polyps (FGPs) and increase the size of existing FGPs [2, 3, 4]. Furthermore, PPIs have been associated with cases of bleeding from fundic gland polyp associated with PPI (PPI‐FGP) and some cases of PPI‐FGP can undergo neoplastic transformation [5, 6]. Therefore, the diagnosis of PPI‐FGP is clinically important and requires follow‐up endoscopy. PPI‐FGPs are generally larger and more edematous than FGPs and exhibit an enlargement of fundic gland cysts just below the surface of the gastric mucosa [2, 3, 4]. However, we have also observed PPI‐FGPs that are smaller, and therefore more difficult to diagnose endoscopically based on an edematous appearance.

In this regard, image‐enhanced endoscopy can be used for the diagnosis of various gastroenterological lesions. Methodologies used in image‐enhanced endoscopy such as texture and color enhancement imaging (TXI, Olympus Medical Systems Corporation, Tokyo, Japan), narrow‐band imaging (NBI), linked color imaging, and blue laser imaging are now routinely applied in the clinic practice [7, 8, 9, 10, 11, 12, 13, 14, 15, 16, 17, 18, 19, 20, 21, 22, 23]. The recent application of TXI to image‐enhanced endoscopy has identified differences in mucosal color and structure between normal and diseased tissues, which in turn has greatly facilitated the detection of lesions [23]. This is underscored by the success of TXI in the diagnosis of gastroesophageal and colorectal neoplasms, mucosal atrophy, and ulcerative colitis [7, 8, 9, 10, 11, 12, 13, 14].

We previously identified a region within the edematous area that has a gray appearance upon endoscopy and is specifically associated with PPI‐FGP in Japanese medical books [24]. We hypothesized that this gray may facilitate endoscopic diagnosis of PPI‐FGP and allow the differentiation of PPI‐FGP from FGP. To date, however, there are reports of analyses that examine whether this gray region is associated with polyp pathology. In addition, the relative visibility of this region following white light imaging (WLI) and TXI has not been evaluated.

In this study, we define the gray region as the ‘gray color sign’ (GCS) and investigate whether the visibility of GCS is improved in TXI compared to WLI. In addition, we investigate the relevance of GCS to PPI‐FGP pathology.

## 2 Methods

### 2.1 Study Design and Patients

In a single‐center prospective clinical study, we evaluated whether GCS captured during endoscopy was more readily detected by TXI‐1, TXI‐2, and NBI compared to WLI. We took biopsies of GCS areas within the registered lesions to examine GCS histology. We enrolled patients who underwent esophagogastroduodenoscopy (EGD) with WLI, TXI‐1, TXI‐2, and NBI from April 2021 to October 2022 at our hospital, and who received a histopathological diagnosis of PPI‐FGP. The inclusion criteria for enrolment were: patients over 20 years old with a history of taking PPIs/PCABs for at least 6 months and were continued to take it to the current time. Endoscopies were performed for a variety of reasons, including the presence of abdominal pain, medical check‐ups, gastroesophageal reflux disease symptoms, and follow‐up appointments for all of these issues. We excluded patients with a history of gastrectomy or advanced gastric cancer, and those ineligible for endoscopy due to serious primary diseases such as heart, hepatic, or respiratory failure [25].

### 2.2 EGD Procedure

We used GIF‐H290Z, GIF‐XZ1200 endoscopes, the EVIS X1, and CV‐1500 systems (Olympus Medical Systems Corporation) [25]. All endoscopic examinations were performed by expert endoscopists (Yoichi Akazawa, Tsutomu Takeda, and Hiroya Ueyama). Imaging was first performed using WLI, which was followed by TXI‐1, TXI‐2, and NBI. The WLI image enhancement level was set to B6, and the TXI image enhancement level was set to moderate. Three expert endoscopists (Yoichi Akazawa, Tsutomu Takeda, and Hiroya Ueyama) assessed and discussed WLI, TXI, and NBI images until all were in agreement for each image. JPEG images were downloaded from a server in a lossless format; each image file was about 100 kB, with a pixel array of 640 × 510 and 24‐bit color [25].

### 2.3 Definition and Histopathological Analysis of PPI‐FGP, GCS, and Black Spots

**FIGURE 1:**
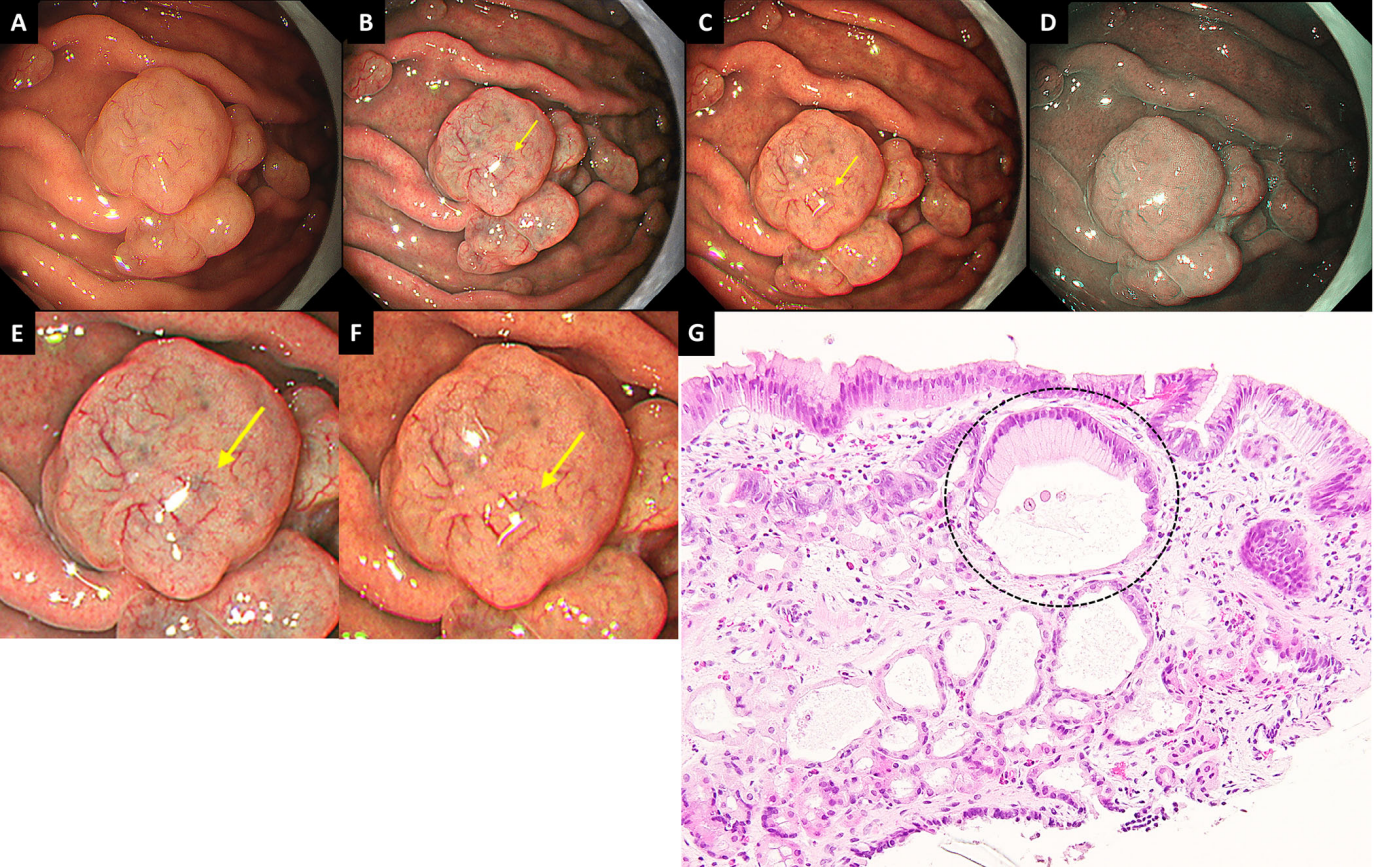
Definition and histopathological analysis of gray color sign. (A) WLI. Representative endoscopic image of PPI‐FGP. GCS was not clearly visible. (B) TXI‐1. The visibility of GCS (yellow arrow) improved compared to WLI. (C) TXI‐2. The visibility of GCS (yellow arrow) improved compared to WLI. (D) NBI. GCS was not observed. (E) Magnified image of Figure 1B. (F) Magnified image of Figure 1C. (G) The histological findings of a biopsy specimen of GCS. Enlargement of fundic gland cysts (mixture type) was observed just below the surface. PPI‐FGP, fundic gland polyp associated with proton pump inhibitor; GCS, gray color sign; WLI, white light imaging; TXI‐1, texture and color enhancement imaging mode1; TXI‐2, texture and color enhancement imaging mode2; NBI, narrow band imaging; FGCs, fundic gland cysts.

**FIGURE 2:**
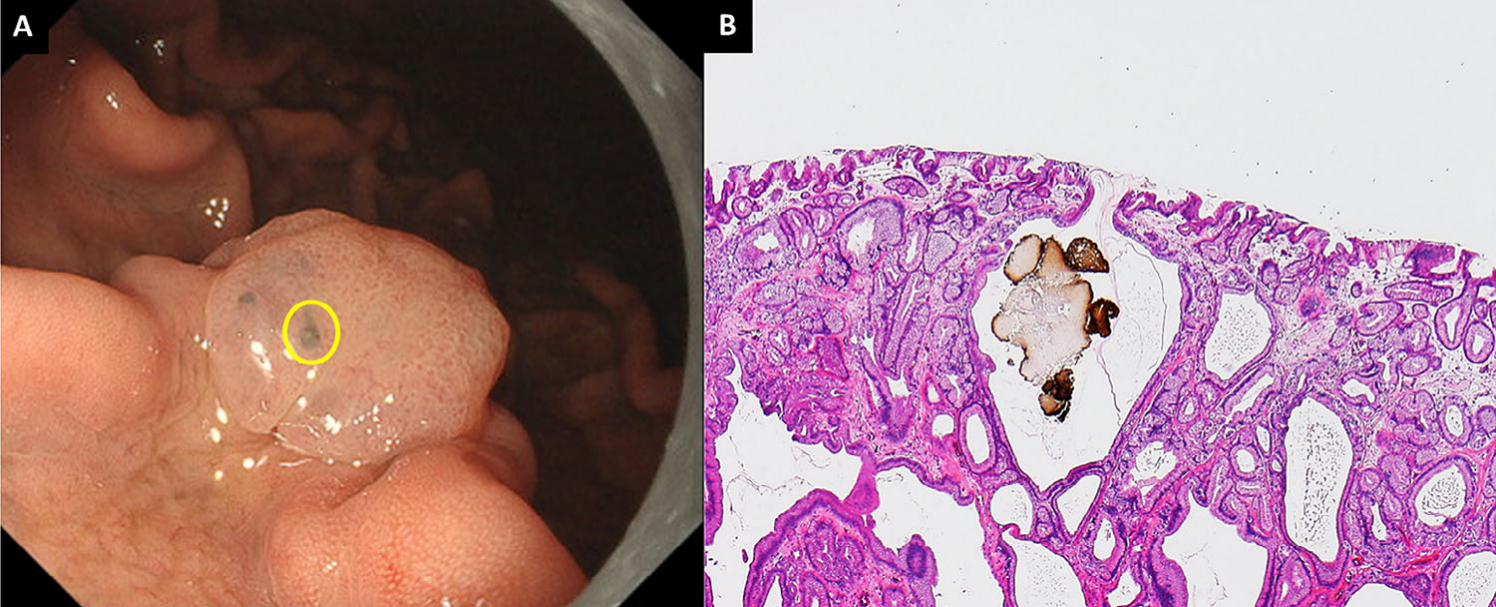
Endoscopic features and histopathological findings of black spots. (A) BSs were seen in the PPI‐FGP. (B) Parietal cell protrusions, fundic gland cysts, and brownish pigmentation in fundic gland cysts (Hematoxylin and Eosin [H&E] staining, 100×). BSs, black spots; PPI‐FGP, fundic gland polyp associated with proton pump inhibitor.

A PPI‐FGP diagnosis was based on the presence of lesions with at least one of the following histopathological findings following biopsy: parietal cell protrusion, foveolar cell hyperplasia, or enlargement of fundic gland cysts [26, 27, 28, 29, 30, 31]. GCS was defined as a mottled or circular gray translucent finding in PPI‐FGP (Figure 1A–F) [24]. Black spots (BSs) were defined as black pigmentation in gastric mucosa by conventional endoscopic WLI (Figure 2A) [3]. BSs have been known to be composed of brownish substances and eosinophilic materials in fundic gland cysts, which are thought to result from the accumulation of secretion from the lining cells of fundic gland cysts [3, 24, 32].

Following the isolation of biopsies from the GCS region, we analyzed the following characteristics: the presence of parietal cell protrusion, foveolar cell hyperplasia, histopathological classification of enlarged fundic gland cysts just below the surface (fundic gland type, foveolar type, and mixture type), and presence of brownish substances and eosinophilic materials in fundic gland cysts.

### 2.4 Evaluation of GCS Visibility

Ten endoscopists (five experts and five trainees) compared GCS visibility between TXI‐1, TXI‐2, NBI, and WLI. Images were presented randomly against a dark background in PowerPoint. WLI, TXI‐1, TXI‐2, and NBI images were shown next to each other. Endoscopists were blinded with respect to clinical data and image capture dates. All endoscopists were not familiar with the characteristics of TXI and GCS and had not received any prior guidance. Combined score classifications were as follows: 40 points, improved visibility; 21–39 points, comparable to WLI; <20 points, decreased visibility. The intra‐class correlation coefficient (ICC) was used to indicate inter‐ and intra‐rater reliability [25].

### 2.5 Color Analysis

After processing in Adobe Photoshop CC 2019, images were assessed using L* a* b* (L* = light/dark; a* = red/green; b* = yellow/ blue) color scores in a Commission Internationale de l'éclairage (CIE) LAB color space system [33]; this method was described previously [34]. A region of interest (ROI; 20 × 20 pixels) was for both gray and non‐GCS areas in PPI‐FGP images. Color values (*L, a, b*) within the ROI and their mean [A1] were quantified from a histogram analysis. L, a, and b represent the lightness, green‐red axis, and blue‐yellow axis, respectively, in the CIELAB color space. The L, a, and b values were converted to the CIELAB color space using the following transformations: L* = L/256 × 100; a* = a – 128; b* = b – 128 [35, 36]. Color differences (ΔE*) between pixel values within the ROI were calculated using the Euclidean distance formula in the CIELAB color space to evaluate the perceptual difference between color images. The formula used was: ΔE = [(ΔL) 2+(Δa*)2+(Δ b*) 2] 1/2).

### 2.6 Statistical Analysis

Differences in visibility scores rated by trainees, experts, ΔE* and L* a* b* color values between images were evaluated for statistical significance (*p* < 0.01) using the Wilcoxon rank sum test. Inter‐ and intra‐rater reliability was tested using the ICC with 95% confidence intervals (CIs). Inter‐rater reliability for interval, ordinal, and ratio variables was assessed using the ICC where multiple coders were involved [37]. Reliability was classified as follows: “perfect” when ICC was 1.0, “excellent” when >0.81, “substantial” when 0.80–0.61, “moderate” when 0.60–0.41, “fair” when 0.40–0.21, and “slight” when <0.20 [38, 39]. The use of 10 assessors ensured that the sample size was sufficient to generate reliable estimates. A minimum acceptable reliability coefficient (ρ₀) of 0.7 and an expected reliability coefficient (ρ₁) of 0.8 were established as benchmarks. A difference at α = 0.05 and β = 0.2 was detected using a sample size of 23 [40]. All statistical analyses were performed using SAS v. 9.4 (SAS Institute, Cary, NC, USA).

## 3 Results

### 3.1 Baseline Characteristics

We enrolled 19 patients, from whom a total of 23 PPI‐FGP lesions were evaluated (Table 1). The mean age was 65.5 years (range: 51–84); 11 patients were male and eight were female. All patients were taking PPIs or PCABs, with esomeprazole the most commonly taken PPI (eight cases) followed by PCABs taken by four patients. The administration period of PPIs or PCABs was at least 15 months for all patients, with a median of 74 (±45.7) months. The most common reason for taking PPIs or PCABs was reflux esophagitis (eight cases), although they were also taken to prevent ulcers induced by other agents such as anti‐thrombotic drugs. The median diameter of PPI‐FGPs was 9 mm (range: 5–20). Atrophic gastritis was rare in this patient group and almost all patients were *Helicobacter pylori*‐ negative.

**TABLE 1:** Baseline characteristics (*n* = 19).

Male/female	11	8	
Age	51–84 (65.5)		
Smoking history (+/−)	10	9	
Alcoholic history (+/−)	12	7	
PPI/PCAB types			
Rabeprazole	1		
Esomeprazole	8		
Lansoprazole	6		
Vonoprazan	4		
PPI/PCAB treatment length			
Range (month)	15–179		
Median (SD)	74 (± 45.7)		
Administration period of PPIs or PCABs			
Reflux esophagitis	7		
Functional dyspepsia	3		
Antithrombotic drug	6		
Steroid	2		
NSAIDs	1		
Diameter of PPI‐FGPs			
Range (mm)	5–20		
Median (SD)	9 (± 4.15)		
Atrophic gastritis (none / closed / open)	18	1	0
*H. pylori* infection status (positive /post eradication / uninfected)	0	1	18

Abbreviations: NSAIDs, non‐steroidal anti‐inflammatory drugs; PCAB, potassium‐competitive acid blocker; PPI, proton pump inhibitor; PPI‐FGPs, fundic gland polyps associated with proton pump inhibitor; SD, standard deviation.

### 3.2 Histopathological Features of PPI‐FGP and GCS

The histopathological features of PPI‐FGP and GCS are shown in Table 2. Both parietal cell protrusion and foveolar cell hyperplasia were found in almost all lesions (91.3% [21/23] and 95.7% [22/23], respectively). Enlargement of fundic gland cysts and fundic gland cysts just below the surface were observed in all lesions. The gland subtypes were fundic (91.3%; 21/23), foveolar (65.2%; 15/23), and mixed (78.2%; 18/23). No lesions were exclusive of the fundic gland type, and all lesions contained either foveolar type, mixed type, or both. No brownish substances or eosinophilic materials were found in any of the fundic gland cysts.

**TABLE 2:** Histopathological features of PPI‐FGP and gray color sign.

		*n* = 23
	(+)	(−)
Parietal cell protrusion, *n* (%)	21 (91.3)	2 (8.7)
Foveolar cell hyperplasia, *n* (%)	22 (95.7)	1 (4.3)
Enlargement of fundic gland cysts, *n* (%)	23 (100)	0 (0)
Fundic gland type, *n* (%)	21 (91.3)	2 (8.7)
Foveolar type, *n* (%)	15 (65.2)	8 (34.8)
Mixture type, *n*. (%)	18 (78.2)	5 (21.8)
Enlargement of fundic gland cysts just below the surface, *n* (%)	23 (100)	0 (0)
Brownish substances and eosinophilic materials in fundic gland cysts, *n* (%)	0 (0)	23 (100)

Abbreviation: PPI‐FGP, fundic gland polyp associated with proton pump inhibitor.

### 3.3 Evaluation and Scoring of GCS Visibility

Comparisons of the performance of TXI‐1, TXI‐2, or NBI and WLI with regard to GCS visibility were carried out by both trainee and expert endoscopists (Table 3). Compared to WLI, all endoscopists reported that GCS visibility was improved by TXI‐1 (82.6%; 19/23) and TXI‐2 (86.9%; 20/23). Trainees reported improved visibility for TXI‐1 (95.6%; 22/23) and TXI‐2 (86.9%; 20/23), and experts reported improved visibility for TXI‐1 (82.6%; 19/23) and TXI‐2 (86.9%; 20/23). None of the observers reported an improvement in GCS visibility when NBI was used.

**TABLE 3:** Evaluation of texture and color enhancement imaging and narrow‐band imaging for visibility.

	All endoscopists			Experts			Trainees		
Visibility n (Δ%)	TXI‐1	TXI‐2	NBI	TXI‐1	TXI‐2	NBI	TXI‐1	TXI‐2	NBI
Improvement	19 (82.6)	20 (86.9)	0	19 (82.6)	20 (86.9)	0	22 (95.6)	20 (86.9)	0
Equivalent	4 (17.3)	3 13.0)	5 (17.0)	4 (17.3)	3 (13.0)	8 (34.8)	1 (4.4)	3 (13.0)	7 (30.4)
Decrease	0	0	18 (83.0)	0	0	15 (65.2)	0	0	16 (69.6)

Abbreviations: NBI, narrow‐band imaging; TXI, texture and color enhancement imaging; TXI‐1, TXI mode1; TXI‐2, TXI mode2.

The visibility scores reported by experts, trainees, and all endoscopists for TXI and NBI compared with WLI are shown in Table 4. Total visibility scores were as follows: TXI‐1 (44.9), TXI‐2 (42.9), and NBI (17.4) for all endoscopists; TXI‐1 (22.7), TXI‐2 (21.8), and NBI (8.5) for trainee endoscopists; TXI‐1 (22.2), TXI‐2 (21.1), and NBI (8.8) for expert endoscopists. Total visibility scores for all endoscopists were significantly higher using TXI‐1 and TXI‐2 compared with NBI (*p* < 0.01). The TXI‐1 and TXI‐2 visibility scores reported by trainees were not significantly different from those reported by experts.

**TABLE 4:** Visibility scores and evaluation of texture and color enhancement imaging for inter‐rater reliability of experts, trainees, and all endoscopists (mean ± SD).

	All endoscopists (N:10)	Experts (N:5)	Trainees (N:5)	Experts versus Trainees (*p*‐value)	TXI‐1 versus NBI (*p*‐value)	TXI‐2 versus NBI (*p*‐value)	TXI‐1 versus TXI‐2 (*p*‐value)
GCS	Score						
TXI‐1	44.9 ± 4.3	22.2 ± 2.3	22.7 ± 2.1	0.5	<0.01	<0.01	0.37
TXI‐2	42.9 ± 4.6	21.1 ± 2.3	21.8 ± 2.4	0.32			
NBI	17.4 ± 4.0	8.8 ± 2.8	8.5 ± 1.9	0.67			

Abbreviations: GCS, gray color sign; NBI, narrow band imaging; TXI‐1, texture and color enhancement mode1; TXI‐2, texture and color enhancement mode2.

### 3.4 Inter‐Rater Reliability

Table 5 shows the comparison of inter‐rater reliability scores (reported as ICC values) for each rater subgroup and for all endoscopists. The comparison of TXI‐1 and TXI‐2 to WLI by trainees gave ICC scores of 0.858 and 0.889, respectively. For experts, the corresponding scores were 0.864 and 0.817, while for endoscopists the scores were 0.931 and 0.927. Since all scores were >0.81, we conclude that the inter‐rater reliability with regard to comparisons of TXI‐1 and TXI‐2 to WLI was “excellent” for all rater subgroups [25].

**TABLE 5:** Evaluation of texture and color enhancement imaging for inter‐rater reliability of experts, trainees, and all endoscopists.

GCS	All endoscopists (*n* = 10)	Experts (*n* = 5)	Trainees (*n* = 5)
ICC (2.1)^a^	Score		
TXI‐1	0.931	0.864	0.858
TXI‐2	0.927	0.817	0.889

Abbreviations: GCS, gray color sign; ICC, the intra‐class correlation coefficient; TXI‐1, texture and color enhancement mode1; TXI‐2, texture and color enhancement mode2.

^a^ “perfect”; 1.0, “excellent”; 0.99–0.81, “substantial”; 0.80–0.61, “moderate”; 0.60–0.41, ‘‘fair; 0.40–0.21, “slight”; <0.20.

### 3.5 Objective Evaluations

**FIGURE 3:**
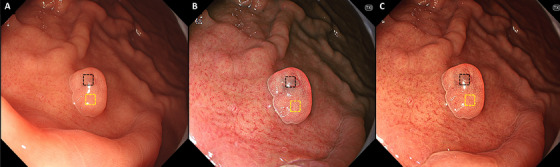
Representative endoscopic images using WLI, TXI‐1, and TXI‐2 with ROIs. (A) WLI. (B) TXI‐1. (C) TXI‐2 with the region of interest (ROIs, 40×40 pixels). ROIs were set in non‐GCS (yellow box), GCS (black box), and the same positions in all three modes (WLI, TXI‐1, TXI‐2) were selected. WLI, white light imaging; TXI‐1, texture and color enhancement imaging mode1; TXI‐2, texture and color enhancement imaging mode2; ROIs, region of interests; GCS, gray color sign.

Representative endoscopic images using WLI, TXI‐1, and TXI‐2 with ROIs are shown in Figure 3. L* a* b* color values for non‐GCS (yellow box) and GCS (black box) were calculated. Table 6 shows the comparison of L* a* b* values for GCS and non‐GCS images within the PPI‐FGP. There was a significant difference in the a* b* values (*p* < 0.01) for GCS when TXI‐1 was compared to WLI. L* values were significantly lower when TXI‐2 was compared to WLI. ΔE* values were as follows: WLI, 12.0 ± 4.3; TXI‐1, 22.3 ± 7.8; TXI‐2, 25.9 ± 7.0. Significant differences in ΔE* were observed for both TXI‐1 and TXI‐2 compared to WLI (*p* < 0.01). There was no significant difference in ΔE* between TXI‐1 and TXI‐2.

**TABLE 6:** Objective evaluations using L*, a*, b* color values and color difference (ΔL*, Δa*, Δb*, ΔE*; mean ± SD).

					*p*‐value		
L* a* b* values		WLI	TXI ‐1	TXI‐2	WLI versus TXI ‐1	WLI versus TXI ‐2	TXI‐1 versus TXI‐2
Non‐GCS	L*	70.5 ± 7.4	73.7 ± 8.7	72.5 ± 7.2	0.18	0.36	0.61
	a*	30.1 ± 8.8	21.8 ± 13.3	29.1 ± 9.4	0.02	0.72	0.04
	b*	34.2 ± 4.9	23.3 ± 6.4	36.9 ± 6.8	<0.01	0.13	<0.01
GCS	L*	62.0 ± 7.5	59.2 ± 11.4	51.1 ± 7.0	0.33	<0.01	<0.01
	a*	32.9 ± 6.3	15.7 ± 11.8	32.7 ± 11.1	<0.01	0.92	<0.01
	b*	34.2 ± 4.5	18.4 ± 9.4	30.7 ± 5.4	<0.01	0.02	<0.01
	ΔL*	8.5 ± 5.7	14.0 ± 10.4	21.1 ± 7.0	0.34	<0.01	<0.01
	Δa*	−2.7 ± 6.2	6.2 ± 11.0	−3.5 ± 9.3	<0.01	0.74	<0.01
	Δb*	0.0 ± 5.4	6.3 ± 8.6	4.9 ± 7.7	<0.01	0.02	0.57
	ΔE*	12.0 ± 4.3	22.3 ± 7.8	25.9 ± 7.0	<0.01	<0.01	0.1

Abbreviations: ΔE*, color difference; GCS, gray color sign; NBI, narrow band imaging; TXI‐1, texture and color enhancement mode1; TXI‐2, texture and color enhancement mode2; WLI, white light imaging.

## 4 Discussion

In this study, we found that TXI‐1 and TXI‐2 were both superior to WLI with regard to visualization of PPI‐FGP‐associated GCS; this was the case whether observations were made by trainee or expert endoscopists. In addition, our histopathology analysis revealed enlargement of fundic gland cysts in the GCS and confirmed that this is an endoscopic finding specific to PPI‐FGP.

For this study, we used ‘GCS’ to define a region with a gray appearance within the edematous PPI‐FGP. Histopathological analysis of PPI‐FGP showed biopsy in all lesions, with foveolar type and mixture type of fundic gland cysts more frequently seen than in normal FGP (Figure 1E). These results were similar to the previously reported histopathological analysis of PPI‐FGP [31]. In addition, Yao et al. reported that in normal FGP, the formation of enlargement of fundic gland cysts is thought to be the result of obstruction of mucus drainage produced by mucous neck cells in the deep mucosa [41]. GCS was defined as the mottled or circular gray translucent finding in PPI‐FGP, and GCS was thought to be derived from the enlargement of fundic gland cysts just below the surface by comparison between endoscopic and histopathological finding (Figure 1B,G) [24]. Therefore, GCS was considered an endoscopic finding specific to PPI‐FGP.

Black pigmentation observed in the gastric mucosa by conventional endoscopic WLI is referred to as ‘BSs’ [3, 32]. These structures within fundic gland cysts are composed of brownish substances and eosinophilic materials [3, 32]. To begin with, BSs are not an endoscopic finding specific to PPI‐FGP but are also observed in the gastric mucosa of the fundic gland region, normal FGP, gastric type adenoma, and gastric adenocarcinoma of fundic gland type [42]. If present in the deep areas of FGP, BSs may appear gray on endoscopic images, which would make it challenging to distinguish between BSs and GCS [3, 24, 32]. However, we find here that histology‐confirmed GCS areas did not contain brownish substances or eosinophilic materials in fundic gland cysts in all lesions. Therefore, we conclude that GCS and BSs are distinct entities.

There have been several reports on the usefulness of TXI in gastric lesions. Ishikawa et al. reported that gastric mucosal atrophy is more readily visualized using TXI‐1 compared to WLI and that TXI‐1 emphasizes blue and white tones characteristic of gastric mucosal atrophy [10]. In our current study, we found that TXI‐1 and TXI‐2 improve the visibility of GCS compared to WLI. Specifically, the *Δa* and the *Δb* values were significantly larger in TXI‐1, leading us to conclude that the color enhancement functions caused a significant difference in *ΔE*. Compared to WLI, the *ΔL* value was significantly larger in TXI‐2, and as a result, the contrast with the surrounding area is clearer, which likely explains the improved visibility. Overall, it appears that the improved performance of TXI‐2 over WLI may be due to brightness enhancement functions. This seems reasonable in light of previous reports on other types of lesions using TXI‐1 and TXI‐2 [7–14, 23, 25]. In addition, Sato et al. reported that the structure enhancement function of TXI strengthens color and brightness enhancement [23]. We infer that this structure enhancement function might also contribute to the improvement of GCS visibility. Furthermore, in this study, there was no significant difference in the visibility of GCS between TXI‐1 and TXI‐2, and both are considered to be useful.

In contrast to TXI, we found that GCS detection was poor when NBI was used. This is because NBI improves the visibility of blood vessels and epithelial architecture, and neither of these features is present in GCS [43].

Our study has several limitations, including the enrolment of only a small number of patients from a single center, selection bias in the lesions, and potential observer bias due to the subjective assessment of visibility. To mitigate these issues, we introduced an objective quantitative analysis with color differences for each image. We did not determine the frequency of GCS occurrence in PPI‐FGP in this study and did not investigate whether TXI would be useful for detecting PPI‐FGP in clinical practice. These points should be addressed in the future and would benefit from the design and execution of prospective multi‐center studies. Therefore our data may not directly address the utility of TXI in a real‐world clinical setting. Randomized studies of larger patient cohorts with further in‐depth histological analyses, will be required to confirm our results.

In conclusion, our research has clarified the pathological features of GCS and has established the superiority of TXI over WLI for the visualization of GCS. In addition, since GCS is present in PPI‐FGP regardless of the lesion size, the use of TXI may facilitate the diagnosis of PPI‐FGP in the future.

## Ethics Statement

Approval of the research protocol by an Institutional Reviewer Board: The present study was approved by the ethics committee of Juntendo University Hospital (No. 20–347) and was performed according to the tenets of the Declaration of Helsinki.

## Consent

All patients provided written, informed consent to participate in this study.

## Conflicts of Interest

The authors declare no conflicts of interest.

## Clinical Trial Registration

This study is registered at the University Hospital Medical Research Network (number UMIN000045323).

## Supporting information



Figure S1
