# Supplementary figures and images for: Visibility Evaluation of Fundic Gland Polyp Associated With Proton Pump Inhibitor in Texture and Color Enhancement Imaging
Source: DEN Open. 2025 May 22;6(1):e70147. doi: 10.1002/deo2.70147 (PMC12097350; doi:10.1002/deo2.70147)

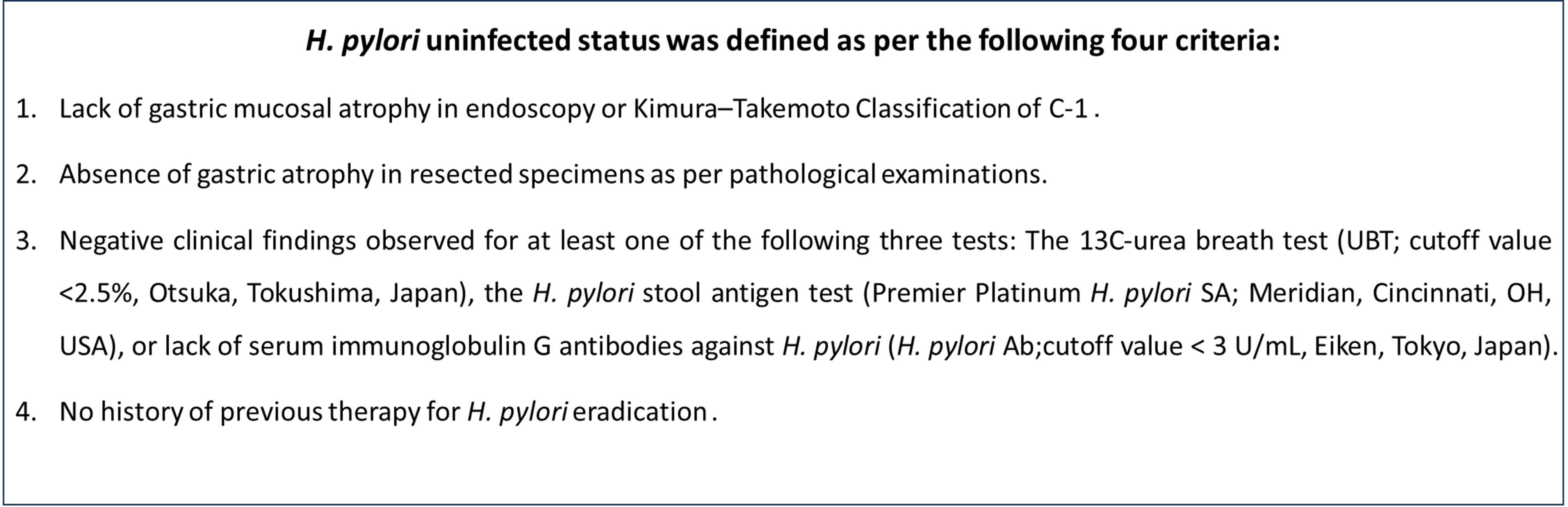

Supplement: Supplementary file 1 — Figure S1 [file DEO2-6-e70147-s001.TIF]
